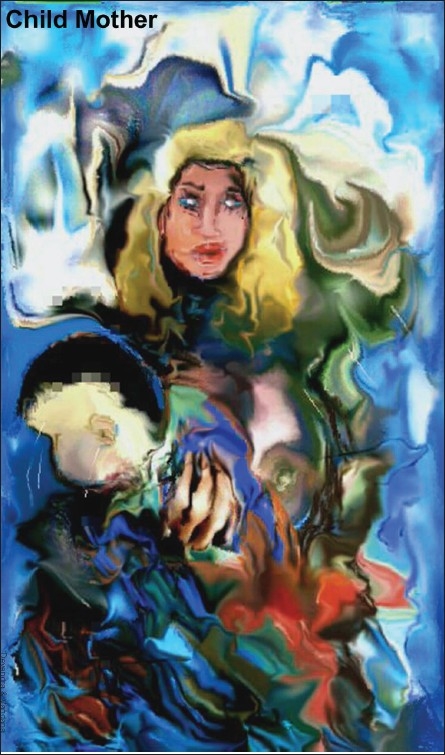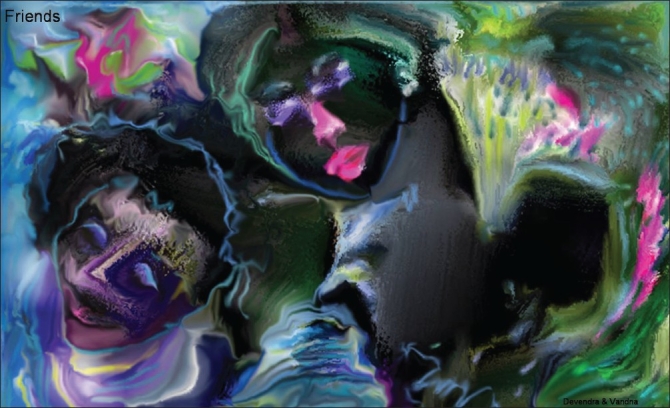# Digital Paintings by Dr. D. K. Gupta

**Published:** 2009